# Effective Connectivity of Depth-Structure–Selective Patches in the Lateral Bank of the Macaque Intraparietal Sulcus

**DOI:** 10.1371/journal.pbio.1002072

**Published:** 2015-02-17

**Authors:** Elsie Premereur, Ilse C. Van Dromme, Maria C. Romero, Wim Vanduffel, Peter Janssen

**Affiliations:** 1 Lab. voor Neuro- en Psychofysiologie, KU Leuven, Leuven, Belgium; 2 Athinoula A. Martinos Center for Biomedical Imaging, Massachusetts General Hospital, Charlestown, Massachusetts, United States of America; 3 Department of Radiology, Harvard Medical School, Boston, Massachusetts, United States of America; Oxford University, UNITED KINGDOM

## Abstract

Extrastriate cortical areas are frequently composed of subpopulations of neurons encoding specific features or stimuli, such as color, disparity, or faces, and patches of neurons encoding similar stimulus properties are typically embedded in interconnected networks, such as the attention or face-processing network. The goal of the current study was to examine the effective connectivity of subsectors of neurons in the same cortical area with highly similar neuronal response properties. We first recorded single- and multi-unit activity to identify two neuronal patches in the anterior part of the macaque intraparietal sulcus (IPS) showing the same depth structure selectivity and then employed electrical microstimulation during functional magnetic resonance imaging in these patches to determine the effective connectivity of these patches. The two IPS subsectors we identified—with the same neuronal response properties and in some cases separated by only 3 mm—were effectively connected to remarkably distinct cortical networks in both dorsal and ventral stream in three macaques. Conversely, the differences in effective connectivity could account for the known visual-to-motor gradient within the anterior IPS. These results clarify the role of the anterior IPS as a pivotal brain region where dorsal and ventral visual stream interact during object analysis. Thus, in addition to the anatomical connectivity of cortical areas and the properties of individual neurons in these areas, the effective connectivity provides novel key insights into the widespread functional networks that support behavior.

## Abstract 2

Combined fMRI and microstimulation in the macaque brain reveals the functional connectivity of neural clusters in the intraparietal sulcus, a region implicated in motor planning, spatial attention, 3-D vision, and grasping.

## Author Summary

The cortex of primates consists of many areas that are highly interconnected, forming widespread functional networks engaged in specific tasks. Cortical areas frequently consist of submodules, columns, or patches of neurons that share functional properties. The neuronal characteristics of such clusters of neurons are determined by their inputs (i.e., from which neurons they receive information) and outputs (i.e., to which neurons in other brain areas they project), but detailed information about the connectivity of small clusters of neurons is frequently lacking. We applied electrical microstimulation during functional magnetic resonance imaging to chart the connectivity of small patches of neurons in the Intraparietal Sulcus, a brain region that has been implicated in many cognitive operations, such as motor planning, spatial attention, 3-D vision, and grasping. We observed that the three patches of neurons we studied were embedded in very distinct functional networks, covering almost the entire cortex. The network of brain areas connected to each patch could, in turn, explain the properties of the neurons in that patch. Thus, the connectivity of clusters of neurons provides crucial information to understand how functional brain networks support behavior.

## Introduction

Extracellular recording studies have provided detailed information on the properties of individual neurons and neuronal populations during task performance, which can be correlated with [1,2], and even causally related to, behavior [3,4]. However in order to fully understand the function of neurons in any given brain area and how these neurons subserve behavior, one also needs information about their anatomical connectivity, i.e., from which areas these neurons receive information (input) and to which areas they project (output). Anatomical tracer studies provide a general roadmap of connectivity but cannot identify how specific types of visual information are transmitted between different levels in the cortical hierarchy, since most far extrastriate cortical areas are highly heterogeneous and frequently contain specialized modules for different types of visual information or cognitive processes [5–7]. Functional magnetic resonance imaging (fMRI) provides a static bird’s-eye view of cortical activations elicited by specific stimuli or tasks [8], yet this indirect measure of brain activity cannot in itself determine how the different nodes of the network are connected and how information flows between these different nodes.

Electrical microstimulation in monkeys during fMRI (EM-fMRI) allows the study in vivo of how neural systems are connected (i.e., effective connectivity [9–14]) at a scale of patches or clusters of neurons. However, no study has used this approach to investigate the areas in the macaque intraparietal sulcus (IPS), which have been implicated in a large number of cognitive processes such as motor planning, spatial attention, decision, reward, timing, 3-D vision, and even categorization [15–21]. In this study, we wanted to relate function to connectivity by implementing single-cell recordings and EM-fMRI in the anterior lateral bank of the IPS. We first identified patches of neurons encoding the depth structure of objects in the anterior intraparietal area (AIP) and subsequently performed EM-fMRI experiments in these functionally defined patches of neurons. Although neurons in anterior and posterior AIP showed highly similar neuronal selectivity, we observed markedly distinct networks of cortical areas in occipital, parietal, frontal, and temporal cortex when stimulating each of these subsectors; anterior AIP was embedded in a somatomotor network, while posterior AIP was connected to areas involved in object processing. Our results demonstrate that the posterior subsector of area AIP may be a critical site of convergence of dorsal and ventral stream object information.

## Results

### Electrophysiological Recordings and Identification of Stimulation Sites

**Fig 1:**
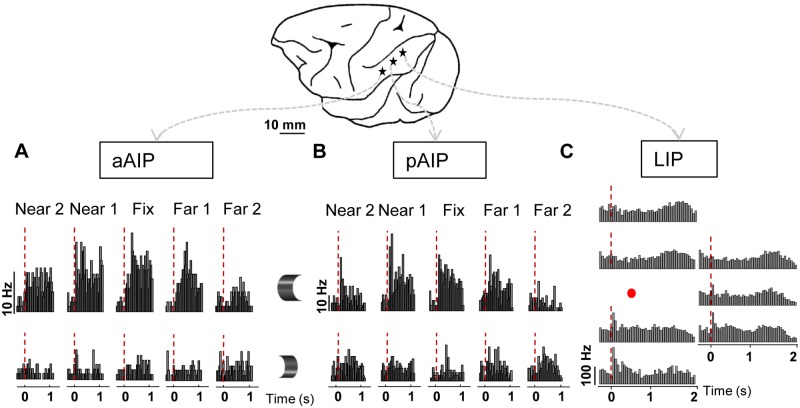
Functional properties of IPS subsectors. A. aAIP. Example neuron showing selectivity for disparity-defined depth-structure (convex versus concave; rows), across different positions in depth (columns). B. Example neuron in pAIP showing selectivity for disparity-defined depth-structure (convex versus concave), across different positions in depth (columns). C. LIP. Example neuron with target-selective responses in a visually guided saccade task. Histograms demonstrate the seven possible target locations, positioned as shown on the screen. The position of the central fixation point is marked with the red dot.

We recorded fMRI-guided single-unit activity (SUA) along the lateral bank of the IPS prior to the EM-fMRI sessions (see overview of EM/recording-positions in S1 Table, S1 Fig. for electrode locations). In two monkeys (M and K), we identified two grid positions in AIP with a high proportion of neurons selective for disparity-defined depth structure (e.g., convex versus concave) (424 recording sites in total, in 22 grid positions), one in anterior AIP (aAIP) and one in posterior AIP (pAIP, [22], Fig. 1A and B). In both subsectors of AIP, a high proportion of the neurons (45% to 65%) preserved their preferences for the depth structure of surfaces (3-D shape) across positions in depth [21], as illustrated by the example neurons in Fig. 1A and B. The aAIP and the pAIP patches contained neurons with highly similar selectivities for disparity-defined curved surfaces. Furthermore, consistent with previous research, we confirmed the presence of object-selective responses (single and multi-unit activity (MUA), monkeys M, K, and C, [23]) and grasping activity in pAIP (monkey C, [24]). As a control, we also recorded spatially selective saccadic activity (SUA and MUA; one-way ANOVA with factor *target position*; *p* < 0.01) in neighboring lateral intraparietal area (LIP) using a visually guided saccade task, in which the saccade target was positioned at seven to ten different locations in the contralateral visual hemifield (monkeys M, K, and T) (example neuron in Fig. 1E; average spike rate during visually guided saccades towards seven different target locations on the screen). Thus, electrophysiological recordings identified three functionally distinct stimulation sites covering two-thirds of the anterior-posterior extent of the lateral bank of the IPS.

### Anterior AIP

**Fig 2:**
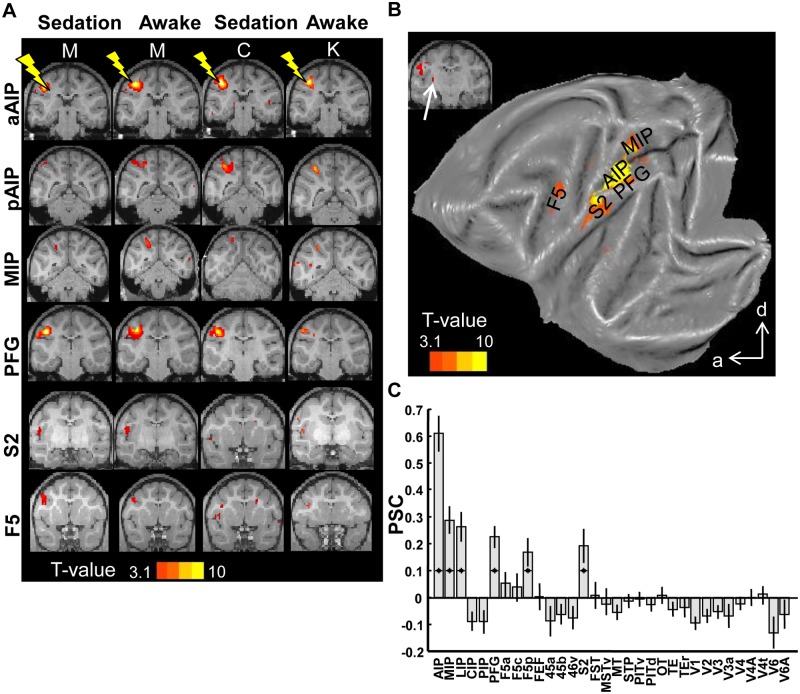
Microstimulation of area aAIP. A. T-score maps for the contrast *EM*-*NoEM*, for individual animals, represented on coronal sections (template anatomy). Leftmost columns show data for monkey M in the sedated and awake states, respectively; rightmost columns show results for monkeys C and K. See files aAIP* in [25]. B. T-score maps for the contrast EM-NoEM, group data, represented on a flat map. a: anterior; d: dorsal. C. Percent signal change calculated in the predefined ROIs. * *p* < 0.05; corrected for multiple comparisons (32 ROIs). Black lines indicate standard error of the mean.

We stimulated aAIP in three different animals (M, K, and C), in ten scan sessions (87 runs × 245 functional volumes, S1 Table for overview, S1 Fig. for electrode locations, data in [25]). Fig. 2A (left column) shows the t-score maps overlaid on coronal sections for monkey M during sedation (contrast *EM* versus *NoEM*; *p* < 0.001, uncorrected; *n* = 17 runs). Focal increases in fMRI-activity were observed in area AIP, consisting of both aAIP and pAIP (Fig. 2A, left column, first and second row), in the anterior lateral bank of the IPS. Furthermore, aAIP-EM elicited significant fMRI activations in the medial bank of the IPS (area MIP), in area PFG in the rostral portion of the inferior parietal lobule, in the most anterior sector of somatosensory area S2 and in ventral premotor cortex (PMv, or area F5; Fig. 2A, left column, rows 2–5). The fMRI activation evoked by aAIP-EM in PMv was located in the posterior bank of the inferior ramus of the arcuate sulcus, comprising both F5p and F5a [26].

Remarkably similar results were obtained when aAIP was electrically stimulated while the animal was awake and performing a task: the same somatomotor network consisting of pAIP, MIP, PFG, S2, and F5 was activated (Fig. 2A, second column, *n* = 41 runs). To quantify the similarity between awake and sedated fMRI-EM in monkey M, we considered 32 pre-defined regions of interest (ROIs) throughout the cortex (see Materials and Methods) and calculated the correlation between the percentage of significant voxels (*p* < 0.001, uncorrected) per ROI in both states (*awake*/*sedated*; data averaged over runs). Awake fMRI-EM correlated strongly (Pearson correlation: 0.78; permutation test, *p* = 0.0002) with fMRI-EM during sedation in the same animal, which allowed us to combine the data from the awake and sedated states in the group analysis. Similarly, the average *t*-value per ROI in the awake state correlated strongly with the average *t*-values in the sedated state (Pearson r = 0.85, permutation test: *p* < 0.0001).

The effects of aAIP-EM were not only similar in the awake and sedated states, but also in different individual animals (compare results obtained from monkey M in first and second column with results from monkeys K-awake and C-sedated in the two rightmost columns of Fig. 2A): although the centers of the activations varied slightly among animals, aAIP-EM consistently elicited fMRI activations in areas AIP (both aAIP and pAIP), MIP, PFG, S2, and F5 in all three animals. The percentage of voxels significantly activated (in the 32 pre-defined ROIs, see Materials and Methods) by aAIP-EM in monkey M was highly correlated with those in monkeys K (Pearson r = 0.60, *p* = 0.01) and C (Pearson r = 0.72, *p* = 0.0042). Moreover, the average *t*-value per ROI in monkey M correlated closely with those obtained in monkeys K (r = 0.36, *p* = 0.04) and C (r = 0.78, *p* = 0.01).

The activation pattern evoked by aAIP-EM was also evident in the group average (fixed effects analysis on all 87 runs; Fig. 2B, average of three monkeys and awake/sedated, *p* < 0.001 uncorrected; see also S2A Fig. for coronal sections). Note that qualitatively similar results were obtained when including the same number of runs per animal (S3 Fig.). An ROI-based analysis showed significant increases in percent signal change (PSC) in areas AIP, MIP, PFG, S2, and F5p during aAIP-EM compared to no-EM (Fig. 2C, *t*-test; *p* < 0.05 corrected for multiple comparisons [32 ROIs]; No-EM is set as the baseline with a zero-value), but not in any of the temporal, occipital or prefrontal ROIs. Note that we did not obtain a significant effect in the ROI of F5a (*t*-test; *p* = 0.37) in the group data, most likely because aAIP-EM activated only a fraction of F5a (Fig. 2B, bottom row). The group data in Fig. 2B also illustrate that the strongest activation in PMv during aAIP-EM was located in area F5p. aAIP-EM did not activate subcortical structures except the putamen (Fig. 2B inset, white arrow), even when the statistical threshold was lowered. Furthermore, the group analysis did not show an effect of aAIP-EM on the contralateral hemisphere (see examples in Fig. 2A, group results in S2A Fig.; PSC in S4A Fig.).

To assess the specificity of our aAIP-EM results, we performed a similar analysis of PSC on ROIs which are not connected to AIP (motor areas F1, F2, F3, F4, F6, and F7; note that early visual areas V1, V2, and V3 in Fig. 2C are also not connected to AIP [27]). We observed no significant increase in PSC in the latter areas during aAIP-EM (S5A Fig.), and the percentage of activated voxels in these areas was very low (S5B Fig.).

To verify the consistency of our results across animals we performed a conjunction analysis on the aAIP-EM data of all individual animals and states (at *p* < 0.05 uncorrected for each animal). The network connected to the anterior subsector of AIP consisted of MIP, PFG, and S2 (S6A Fig.). Although we observed significant AIP-EM–induced activations in area F5 in each animal, the conjunction analysis did not contain F5 due to interindividual differences in the location of the F5 activations (see also Fig. 2A, bottom row) and the very localized character of activations within F5p and F5a. To test the reciprocity of the anatomical connectivity of aAIP, we also stimulated in two target areas of aAIP, namely, PFG (monkey M, 2 sessions, 17 runs) and MIP (monkey K, 14 runs). As expected, PFG-EM activated AIP, S2, and F5p, and MIP-EM activated AIP, PMd, and S2 (S7 Fig.), consistent with the existence of reciprocal connections at this level in the hierarchy of cortical areas [28]. Thus, the most anterior subsector of area AIP—where neurons encoding the depth structure of objects were recorded—is connected to a network of somatosensory and motor areas implicated in reaching and grasping [29–31].

### Posterior AIP

**Fig 3:**
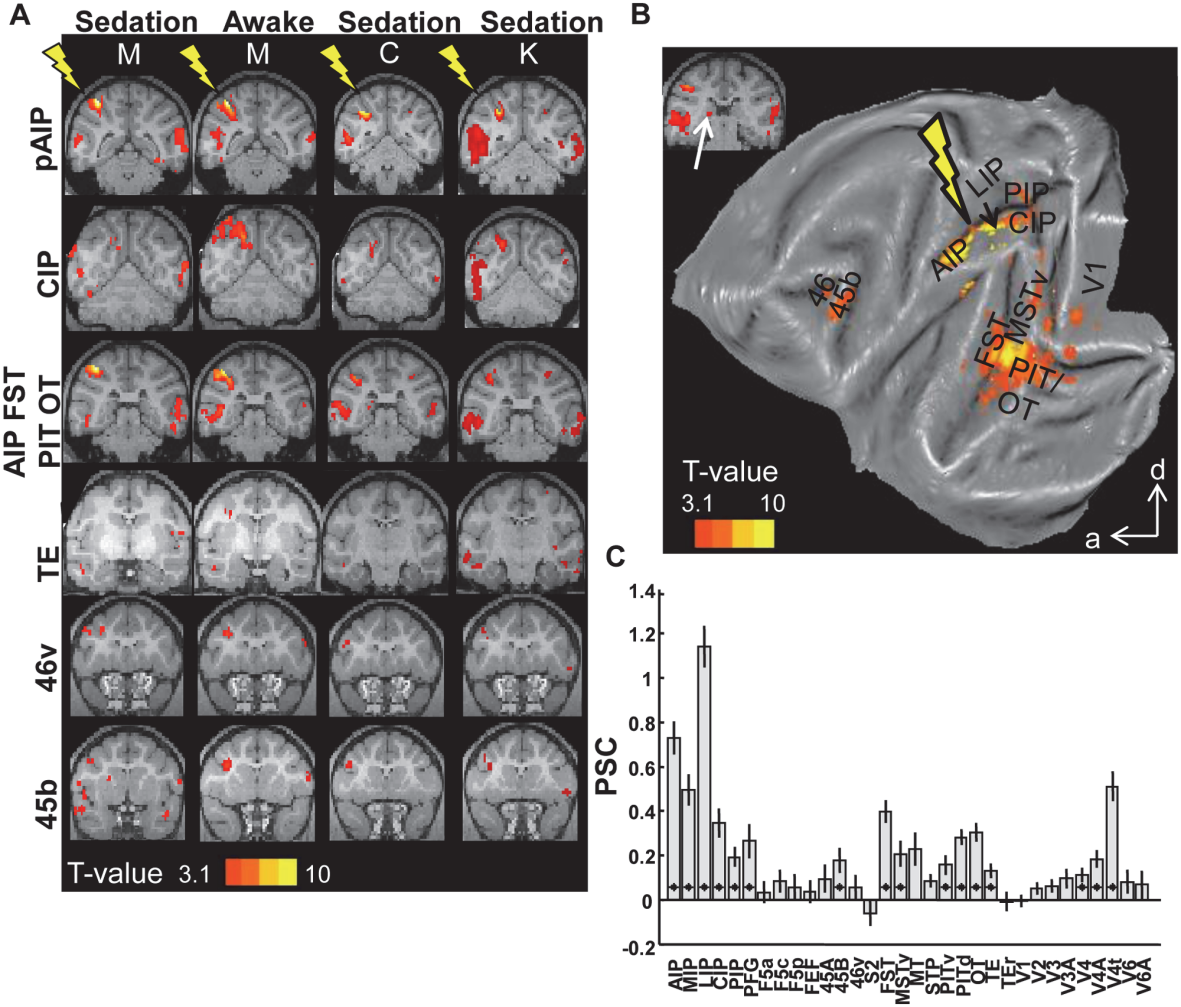
Microstimulation of area pAIP. A. T-score maps for the contrast *EM*-*NoEM* for individual animals, represented on coronal sections (template anatomy). Leftmost columns show data for monkey M in the sedated and the awake state, respectively; rightmost columns show results for monkey C and K. See files pAIP* in [25]. B. T-score maps for the contrast EM-NoEM, group data, represented on a flat map. a: anterior; d: dorsal. C. Percent signal change. * *p* < 0.05; corrected for multiple comparisons (32 ROIs). Black lines indicate standard error of the mean.

Although neuronal characteristics in pAIP were highly similar to those in aAIP, EM of pAIP activated a network of cortical areas that was markedly distinct from the network activated by aAIP-EM (eight scan sessions, 61 runs × 245 functional volumes; see S1 Table for overview per animal; see S1 Fig. for electrode locations; data in [25]). Application of EM to pAIP induced fMRI activations throughout the lateral bank of the IPS; not only in pAIP itself, but also in areas aAIP, LIP, the more posterior subsector of MIP, and in the caudal intraparietal area (CIP) (Fig. 3A, first three rows). In addition, we consistently obtained stimulation-induced activations in the temporal lobe, which was not observed during aAIP-EM (Fig. 3A, third and fourth row): in the lower bank of the superior temporal sulcus (STS), corresponding to the dorsal and ventral part of the posterior inferotemporal cortex (PITd and PITv); the occipitotemporal area (OT); the anterior part of the inferotemporal cortex (TE); the fundus of the STS (FST); and the contralateral STS (Fig. 3A, third row). The pattern of EM-induced fMRI activations was also distinct in the frontal lobe: in contrast to aAIP-EM, stimulating pAIP elicited significant activations in area 45b (in the anterior bank of the inferior ramus of the arcuate sulcus) and in area 46v (Fig. 3A, last two rows). Finally, pAIP-EM also caused scattered activations in and around the lunate and inferior occipital sulcus, corresponding to areas V3 and V4, and even in parts of primary visual cortex V1. As was observed for aAIP-EM, microstimulation of pAIP elicited similar results during awake and sedated experiments in the same animal (Monkey M: Fig. 3A, first and second columns, correlation between the percentage of significantly-activated voxels induced by pAIP-EM during awake versus sedated fMRI: 0.62, *p* = 0.0014). Similar results were obtained in monkeys C (sedated) and K (sedated; Fig. 3A, third and fourth columns; Pearson r = 0.66; permutation test: *p* < 0.0001; and r > 0.30; *p* < 0.05 between C and M, and K, and M). Likewise, the average *t*-values per ROI in monkey M showed a high degree of correlation between the awake and sedated state (r = 0.57, *p* = 0.0018), and between monkeys C and M (r = 0.73; *p* < 0.0001) and K and M (r = 0.52; *p* = 0.003) respectively. The consistency of the pattern of activations elicited by pAIP-EM across animals was confirmed by a conjunction analysis across all three animals, which showed activations in CIP, LIP, pAIP, aAIP, FST, middle temporal area (MT), the ventral part of medial superior temporal area (MSTv), PIT, OT, TE, and 45B (S6B Fig.).

Group data (fixed-effect analysis including all 61 runs; qualitatively similar results were obtained when including the same number of runs per animal, S3B Fig.) of the effect of pAIP-EM are shown in Fig. 3B (see S2B Fig. for coronal sections). In general, pAIP-EM did not evoke significant activations in subcortical structures such as the basal ganglia and the cerebellum. However, in contrast to aAIP, a restricted part of the pulvinar—possibly corresponding to the dorsal pulvinar—was significantly activated by pAIP-EM (Fig. 3B, inset, white arrow). Furthermore, we observed significant activations in posterior parietal cortex (AIP, LIP, CIP in the lateral bank, and MIP and posterior intraparietal area [PIP] in the medial bank of the IPS); in prefrontal areas 45B and part of 46v; and extensive activations in temporal areas FST, PITd/v, OTd, and TE (see S2B Fig. for coronal sections). The PSC was significantly greater than zero in all aforementioned ROIs during pAIP-stimulation compared to no-stimulation (*t*-test; *p* < 0.05, corrected for multiple comparisons [32 ROIs]; Fig. 3C), except for 46v (*p* = 0.37, most likely due to the relatively small part of the area activated by pAIP-EM). In contrast to aAIP, pAIP-EM also elicited contralateral activations in temporal cortex (Fig. 3A, top three rows; S2B Fig.; S4B Fig. for PSC), including areas FST, PITd/v, and OTd.

As for aAIP-EM, a similar analysis of PSC was performed on ROIs which were previously found *not* to be connected to AIP (motor areas F1, F2, F3, F4, F6, and F7). No significant increases in PSC in the latter areas were measured during pAIP-EM (S5A Fig.), and the percentage of activated voxels was very low in the latter ROIs (S5B Fig.). Conversely, the only cortical area found to be connected to AIP as described by tracer studies [27] that was not activated in the current study was the dorsal part of parieto-occipital area V6A.

To quantify the difference in effective connectivity between aAIP and pAIP, we performed an ANOVA on the PSC evoked by EM versus No-EM in all voxels of the 32 predefined ROIs (Materials and Methods). The interaction between the factors Stimulation [EM/NoEM] and area [aAIP/pAIP] was significant (*p* < 0.05) for early visual areas (V1, V2, V3, V3a, V4, V4t, V4A, and V6), temporal areas (OTd, PITd, PITv, TE, FST, MSTv, MT, and STP), prefrontal areas 45b and 45a and parietal areas (S2, MIP, LIP, PIP, and CIP), but not for AIP (see S2 Table). Hence the ROI of AIP was equally activated by aAIP- and pAIP-EM. These results remained essentially unchanged after correcting for the different number of runs (61 for pAIP-EM and 87 for aAIP-EM). Similarly, a conjunction analysis (*p* < 0.01 uncorrected, S2C Fig.) showed that areas AIP, part of area MIP, and a small region in the arcuate sulcus corresponding to F5a were the only areas activated by both aAIP-EM and pAIP-EM.

Taken together, electrical microstimulation in two functionally defined subsectors of area AIP—with very similar neuronal properties and in some cases separated by no more than 3 mm—activated markedly different networks of cortical areas in parietal, temporal, and frontal cortex.

### Area LIP

As a control, we measured the effective connectivity of neighboring area LIP. Data were collected in three monkeys (K, M, and T, see S1 Fig. for electrode locations), in six sessions (60 runs × 245 functional volumes, see S1 Table for overview, data in [25]).

**Fig 4:**
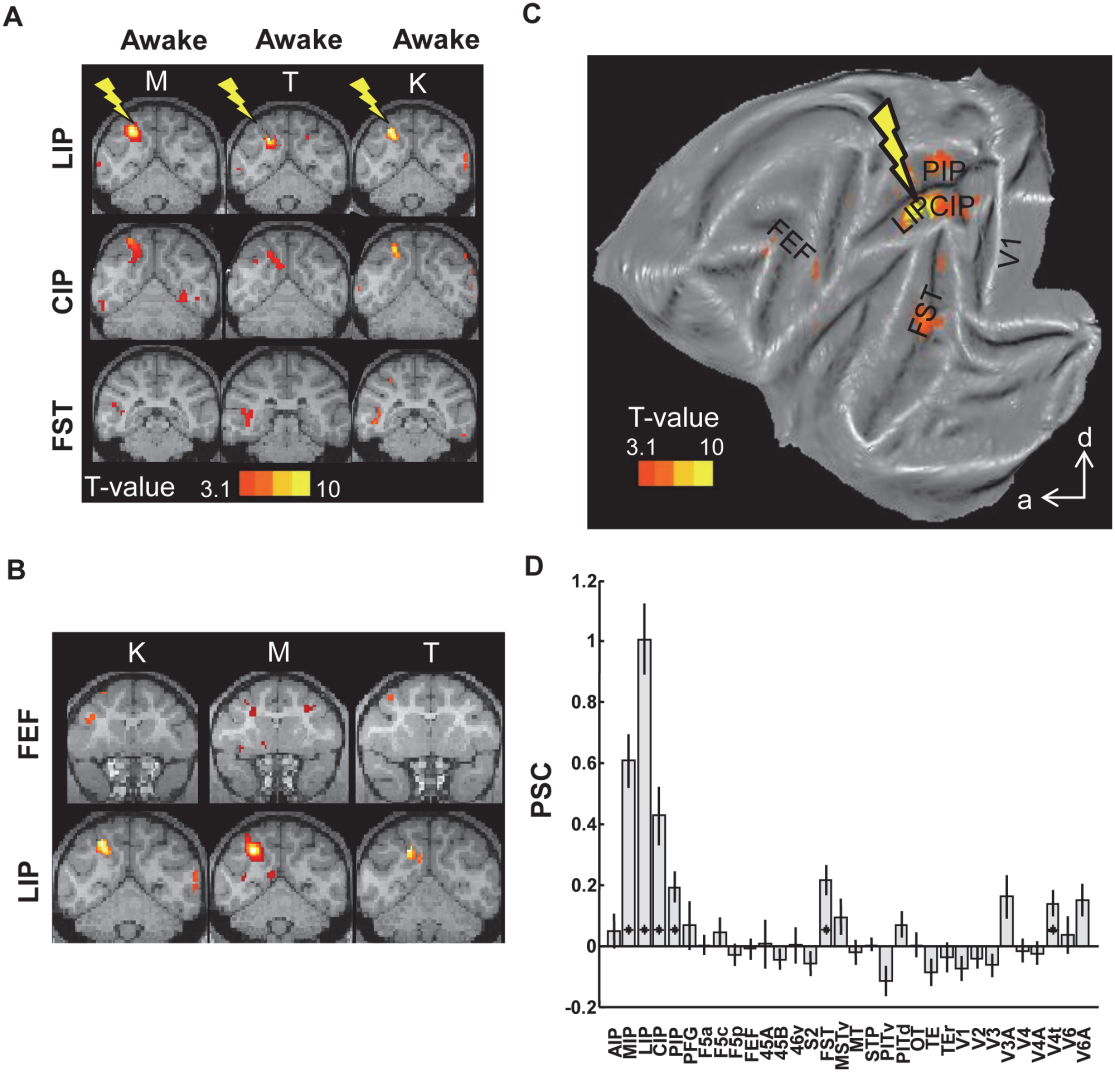
Microstimulation of area LIP. A. T-score maps for the contrast *EM*-*NoEM*, for individual animals, represented on coronal sections (template anatomy). See files LIP* in [25]. B. T-score maps for the contrast *EM*-*NoEM*, data from individual sessions, showing EM-induced FEF activations. C. T-score maps for the contrast *EM*-*NoEM*, group data, represented on a flat map. a: anterior; d: dorsal. D. Percent signal change * *p* < 0.05; corrected for multiple comparisons (32 ROIs). Black lines indicate standard error of the mean.

LIP-EM enhanced fMRI-activations in parietal areas LIP, MIP, CIP, and also in area FST in the fundus of the STS (Fig. 4A, group data in Fig. 4B, S2D Fig.). Furthermore, LIP-EM evoked focal increases in Frontal Eye Fields (FEF)-activity in individual stimulation sessions (three out of six sessions; illustrated in Fig. 4B, upper row). As with the aAIP and pAIP experiments, we obtained comparable results in the sedated and awake states, and similar results in all animals individually (Fig. 4A, correlations between percentage of significant voxels per ROI per monkey/state in 32 predefined ROIs: Pearson r > 0.58, permutation test: *p* < 0.0066; correlations between average *t*-values per ROI per monkey/state in 32 predefined ROIs: r > 0.62, *p* < 0.002). The consistency of the activation patterns elicited by LIP-EM across animals was confirmed by a conjunction analysis (S6C Fig.) across all three animals, which showed activations in CIP, MIP, FST, and V3A.

The group analysis of the LIP-EM experiments revealed a small but significant stimulation-induced increase in activity in FEF (Fig. 4C), in parietal areas LIP, MIP, CIP, and PIP and in temporal area FST. Note that qualitatively similar results were obtained when including the same number of runs per animal (S3C Fig.). Similarly, LIP-EM significantly increased the PSC in parietal areas LIP, MIP, CIP, and PIP, and in temporal area FST (Fig. 4D, *t*-test, *p* < 0.05, corrected for multiple comparisons). In contrast to pAIP-EM, LIP-microstimulation did not modulate the PSC in visual areas V4 and V4A, in temporal areas TE and PITd/v, nor in parietal area AIP or prefrontal area 45B (two-way ANOVA with factors microstimulation [*EM versus no-EM*] and area [*pAIP versus LIP*]; interaction: *p* < 0.05; S2 Table). Thus the IPS sector characterized by the presence of spatially selective saccadic activity (LIP) is effectively connected to a network of cortical areas that only partially overlaps with that of neighboring pAIP.

## Discussion

To our knowledge, our study provides the first causal evidence relating the properties of individual neurons to their effective connectivity in posterior parietal cortex. fMRI-EM in three functionally defined patches of neurons in the lateral bank of the IPS revealed distinct networks of cortical areas in parietal, temporal, and frontal cortex.

Our EM-fMRI results obtained in AIP are highly comparable to earlier work using traditional tracer injections in AIP [27], although the latter authors did not distinguish between anterior and posterior AIP. Our current results are in line with a previous EM-fMRI study, in which FEF-EM showed increased fMRI activations in areas previously found to be anatomically connected to area FEF [11,32,33] (see [34] for review). It is important to emphasize that almost all areas activated during fMRI-EM in aAIP and pAIP are monosynaptically and reciprocally connected with AIP [26,27,35–39]. The only possible exception may be the contralateral PITd/v and OTd activations elicited during pAIP-EM. Conversely, areas that are not directly connected to AIP (e.g., F1–F4, F6–F7, and V1–V3) were also not activated during EM-fMRI in AIP. Only very few brain regions for which AIP connections have been reported were never activated during EM-fMRI: examples include area V6Ad, which is weakly connected to AIP [27], and the cerebellum, which is connected to the possible homologue of AIP in the cebus monkey [40]. Note that with the current resolution of monkey fMRI, it is not possible to make claims about the laminar distribution (supra- versus infragranular) of the connections; hence, it is not possible to make conclusive inferences about feedforward versus feedback connections.

Unlike most tracer studies, we combined extensive single-cell recordings and effective connectivity measurements. However, despite the striking similarity between tracer studies and our results, the crucial advantage of in vivo EM-fMRI (or EM-optical imaging [41]) over tracer studies is that it allows the identification of the connections of specific clusters of neurons (in our case 3-D–shape selective neurons within AIP) with subsectors of other areas (e.g., in frontal cortex), which can then become the target of detailed investigations using (a combination of) single-cell recordings, EM-fMRI, and reversible inactivation during fMRI (see [34]). Thus, in vivo effective connectivity studies furnish the possibility to investigate neural populations for which the inputs and outputs have been accurately identified in the animal under study (possibly even without existing anatomical data), so that the signals can be traced throughout the hierarchy of extrastriate areas from occipital to frontal cortex (see also [11]). Moreover, the same in vivo procedure can be repeated for virtually unlimited numbers of target areas within the same subject. Furthermore, EM-fMRI may also provide important information for the interpretation of the behavioral effects of microstimulation, since a general overview of the effective connectivity of a cortical stimulation site can reveal which downstream areas influence behavior.

Previous tracer studies have shown connections between area LIP and many other (sub)cortical areas such as FEF, 46, parieto-occipital cortex (PO), dorsal prelunate area (DP), 7a, V3, V4, MT, MST, posterior area in inferotemporal cortex (TEO), and superior colliculus [36,37,42,43], in which the ventral stream areas are primarily connected with dorsal LIP while MT/V5 and FEF are primarily connected with ventral LIP [36]. Our current study, however, shows mainly increased activation in temporal area FST, and to a lesser extent in FEF. The difference between our current EM study and previous anatomical tracer studies was not surprising, given the known heterogeneity of area LIP (e.g. [7,44,45]) and the fact that we only stimulated a single site per animal in LIP as a control for pAIP. Moreover, it is conceivable that previous studies (e.g., [46]) have identified our pAIP site, located 9 to 15 mm from the anterior tip of the IPS and with foveal RFs, as anterior LIP (most likely also explaining the connections between area 45B and LIP [38]), whereas we observed 45B activations during pAIP but not LIP stimulation. In addition, our pAIP stimulation site was also located more dorsally in the lateral bank of the IPS, and previous studies have reported that the anterior part of dorsal LIP is strongly connected to ventral stream areas and to area 45B [36].

Other studies [47,48] have investigated effective connectivity in the motor system using a combination of EM and single-cell recordings and have demonstrated that EM can elicit both excitation and inhibition in the target neurons, particularly at higher current strengths. Although marked behavioral effects can be observed with relatively low intensity EM (25–35 μA) in extrastriate cortex [3,49], our pilot experiments showed no reliable fMRI activations when we stimulated with currents below 200 μA (awake) or 1,000 μA (sedated). Since the fMRI signal represents a sum of excitatory and inhibitory activity [50], we probably evoked both effects in our EM-fMRI experiments. It needs to be noted that previous FEF-EM experiments revealed extensive effective connectivity networks with currents below 50 μA in awake animals [11], which could also be obtained by optogenetic stimulation of the same areas [51].

The patterns of activations we observed were highly similar in awake and sedated sessions, similar to lateral geniculate nucleus (LGN)-EM [50]. This observation has important practical implications because it shows that it is possible to chart the connectivity of functional patches of extrastriate neurons in monkeys engaged in single-cell experiments but not accustomed to the scanner environment. However, the strong correspondence between awake and sedated sessions does not necessarily imply that EM-induced activations cannot be stimulus- or task-dependent (e.g., in the Frontal Eye Fields, [11,52]). We did not obtain sufficient data in the current experiment in the awake state to draw definitive conclusions, but future studies should investigate the task-dependency of the fMRI activations evoked by AIP-EM. Importantly, the possibility remains that similarities between the awake and sedated EM-fMRI results are region-specific.

Our effective connectivity results help to understand several anatomical and physiological observations. First, neurons in the posterior part of AIP tend to be more visual, whereas neurons in anterior AIP tend to be more motor-dominant [53], and this visual-to-motor gradient in AIP can now be linked to the connectivity of pAIP (object processing network including the ventral stream) and aAIP (somatomotor network). Our results clearly demonstrate the status of pAIP as a pivotal brain area where dorsal and ventral visual stream interact during object analysis. Before contact with the object, the anterior IPS regions may access information about object identity—which may assist in selecting the appropriate grasp—through these connections with the ventral stream [27,54]. This distinction between pAIP and aAIP, made possible using both effective connectivity and physiological assessments, could not be readily detected in anatomical studies [27]. Future studies should determine to what extent and under which conditions (e.g., immediate versus delayed actions [55,56]) these dorsal–ventral stream interactions become behaviorally relevant.

Secondly, both the aAIP and the pAIP patch contained a high proportion of 3-D–shape selective neurons. The patterns of effective connectivity we observed strongly suggest that 3-D–shape information is transmitted from pAIP to aAIP and subsequently to the motor system. This stream of 3-D–shape information runs along the lateral bank of the IPS and interacts with the ventral stream at the level of pAIP. Note that 3-D–shape selective clusters in AIP are also active during object grasping [57], and that reversible inactivation of these AIP clusters induces a grasping deficit (Verhoef and Janssen, unpublished observations). Therefore our results, in concert with previous findings in AIP, also contribute to our understanding of the organization of the 3-D–shape network.

Finally, the anterior-posterior extents of areas AIP and LIP have been a long-standing controversy, in which anatomical studies have conflicted with physiology studies [42,45,46]. Specifically, the region in the lateral bank of the IPS where spatially selective saccadic activity—interspersed with memory delay-period activity during saccades [7]—can be recorded (i.e., area LIP) is mostly confined to the posterior third of the lateral IPS [42]. In contrast, anatomical studies have claimed, based on the pattern of myelination of LIP [43,45], that LIP occupies a large part of the lateral bank of the IPS. Our data finally suggest a resolution for this issue. The functional properties of individual neurons at the pAIP stimulation site, located 9 to 14 mm posterior to the tip of the IPS, resembled those of aAIP neurons given the presence of strong grasping activity, 3-D–shape selectivity, and the absence of saccadic activity [24,58], and the most posterior tracer injections in AIP in [27] were also located 15 mm posterior to the anterior tip of the IPS. However, the connectivity of pAIP with frontal and temporal areas was strikingly distinct from that of both aAIP and LIP. Therefore, a functional parcellation of the lateral IPS would identify the anterior two-thirds of the lateral IPS, where grasping activity can be recorded, as AIP [53,59,60], and the posterior one-third of the lateral IPS, where spatially selective saccadic activity can be recorded, as LIP. Monkey fMRI studies have also demonstrated a representation of the fovea in the anterior lateral bank of the IPS [61,62], and in humans, two regions in the anterior IPS (DIPSA and DIPSM) are activated more strongly by curved surfaces compared to flat surfaces at different positions in depth [63], which may be homologous to monkey aAIP and pAIP.

Our results are also consistent with human fMRI studies on grasping and object processing. Although considerable differences exist between the human IPS and the macaque IPS (related to, e.g., 3-D structure-from-motion and tool use, [64–67]), the more anterior IPS sectors appear more action-related, whereas the more posterior IPS sectors are more visual [68]. Similarly, resting-state connectivity analysis in humans has indicated that a region in the Lateral Occipital Complex (LOC) responsive to images of hands and tools is selectively connected to the IPS regions involved in action-related processing of hands and tools. It is also noteworthy that distinct patterns of resting-state connectivity can be observed for adjacent seed regions in occipitotemporal cortex [69], similar to the distinct networks we observed when stimulating in different cortical sites that were merely 3 mm apart. Future studies will have to determine the correspondence between functional and effective connectivity as determined with EM-fMRI in the IPS areas, as already achieved in macaque somatosensory cortex [13].

It is remarkable that we observed such distinct networks of cortical areas when stimulating sites that were in some cases separated by no more than 3 mm. Electrical microstimulation at the currents we used undoubtedly activated large numbers of neurons, and most likely not exclusively 3-D–shape, object, or saccade selective neurons. Since the 3-D–shape patches we identified in AIP measured merely 1–2 mm (i.e., one or two grid positions) but were very homogeneous (containing up to 80% 3-D–shape selective neurons [21]), the effect of EM in these patches must have been dominated by the connectivity of 3-D–shape selective neurons. Not surprisingly then, most of the cortical areas connected to the aAIP and pAIP stimulation sites are sensitive to the depth structure of objects [3,5,21,70–75]. Area FST was effectively connected to both pAIP and LIP, consistent with anatomical studies [27,37]. Since FST neurons encode 3-D–shape defined by structure-from-motion (SFM) [76], and in view of the fact that both FST and AIP are selectively activated by SFM-defined 3-D surfaces compared to control stimuli (Mysore, Vogels, Vanduffel, and Orban, unpublished observations), the pAIP-FST connection may be important for the integration of two of the most powerful depth cues, binocular disparity and SFM.

The patterns of connectivity we observed appeared to result mostly from feedforward (e.g., aAIP to F5) and lateral (e.g., aAIP to PFG [27,30]) projections. However, in each stimulation site, EM activated its most likely input areas (feedback): aAIP-EM activated its input area pAIP, pAIP-EM activated LIP and CIP, and LIP-EM activated CIP and V3A, a pattern that is entirely consistent with an earlier anatomical tracer study demonstrating that the main connectivity pattern in the lateral IPS runs from posterior to anterior, from V3A to CIP–LIP–AIP [35]. Visual object information is then send to the motor system (F5p and F5a) and to the somatosensory system (area S2), an area connected to both AIP and F5 where many neurons respond during active hand manipulation of objects but not during passive hand stimulation [77]. Thus, charting the effective connectivity of functionally defined subsectors of areas or patches of neurons in the IPS provides crucial insight into the organization of cortical networks that support behavior.

## Materials and Methods

All experimental procedures were performed in accordance with the National Institute of Health’s Guide for the Care and Use of Laboratory Animals and EU Directive 2010/63/EU, and approved by the Ethical Committee at the KU Leuven. The animals in this study were pair-housed with cage enrichment (toys, foraging devices) at the primate facility of the KU Leuven Medical School. They were fed daily with standard primate chow supplemented with nuts, raisins, prunes, and fruits. The animals received their daily water supply either during the awake experiments, or ad libitum in the cages before and after sedated experiments.

### Subjects

All experiments were performed in four male rhesus monkeys (C: 8 kg; K: 6 kg; M: 5 kg; T: 6 kg). All animals had a custom-made, magnetic resonance imaging (MRI)-compatible headpost and cylinder implanted on the skull using ceramic screws and dental acrylic. All surgeries were performed under isoflurane anaesthesia and sterile conditions. The cylinders were implanted in an oblique orientation (orthogonal to the IPS in monkey C, parallel to the IPS in monkeys M, K, and T) over the IPS at Horsley-Clark coordinates ranging from 10 to 0 P and from 10 to 20 L. In monkey M, the recording cylinder was repositioned before the fMRI-EM experiment in aAIP from an orientation orthogonal to the IPS (S1 Fig., upper row, red arrow) to an oblique orientation parallel to the IPS to allow electrode penetrations parallel to the IPS, targeting the aAIP patch as defined by its neuronal characteristics. Three monkeys (K, M, T) were trained in passive fixation and saccade tasks in a mock fMRI-setup. They were seated in a sphinx position [78] in a plastic monkey chair directly facing an LCD screen (viewing distance: 57 cm). Eye position was monitored at 120 Hz through the pupil position (Iscan, MA, United States). The fourth monkey (C) was scanned only under sedation.

### Electrophysiology

All stimuli were displayed on a CRT monitor (Vision Research Graphics, equipped with P46 phosphor) operating at 120Hz.

**Stereo test**. The stimulus set of the stereo experiment consisted of random-dot stereograms in which depth was defined by horizontal disparity (dot size 0.08 deg, dot density 50%, vertical size 5.5 deg) presented on a grey background [70]. All stimuli were generated using Matlab (MathWorks) and were gamma-corrected. The stimuli in the search test consisted of three types of smoothly curved depth profiles (1, one-half, or one-fourth vertical sinusoidal cycle) together with their antiphase counterparts obtained by interchanging the monocular images between the eyes (disparity amplitude within the surface: 0.5 deg), control stimuli (the monocular images presented to both eyes simultaneously), and flat surfaces at different disparities. Each of the six depth profiles was combined with one of four different circumferential shapes and appeared at two different positions in depth (mean disparity + or—0.5 deg), creating a set of 48 curved surfaces. Ferroelectric liquid crystal shutters (Displaytech) each operating at 60 Hz were used to generate dichoptic presentation. The shutters were synchronized with the vertical retrace of the display monitor. There was no measurable cross-talk between the two eyes [21]. After 200 ms of fixation, the stimulus was presented at the fixation point for 1 s.

In the search test, all stimuli (stereo and control, curved and flat) were presented randomly interleaved at the center of the display and at the fixation plane during passive fixation. Single or multi-unit activity was recorded, and if a site was visually responsive, we isolated single neurons online and tested these neurons in more detail for higher-order disparity selectivity (i.e., selectivity for gradients of disparity) in the position-in-depth test [5]. In this test the stimulus (a combination of a depth profile and a circumferential shape) evoking the highest response in the search test was selected together with its antiphase counterpart, and presented at five different positions in depth ranging from-0.5 degree (near) to +0.5 degree (far) disparity in equal steps.

**Object test**. Previous studies [23,24,58] have characterized pAIP based on the presence of selective visual responses to images of objects presented foveally during passive fixation. The same stimuli as in [23] were used to confirm the presence of object-selective responses in pAIP in three animals (M, K, and C). The stimulus set for the object test consisted of 21 two‐dimensional (2-D) area‐equalized static images of natural and artificial objects, including faces, hands, fruits, branches, and several artificial graspable objects. The presence of object-selective SUA or MUA responses was assessed using a one-way ANOVA (*p* < 0.05).

**Grasping test**. In the visually guided grasping test, a bar attached to a plate was positioned in the monkey’s view. The animal had to rest his right hand on a sensing device in complete darkness for a variable time (inter‐trial interval ITI 3,000–5,000 ms), after which a light inside the object was illuminated, whereupon the monkey had to fixate the object (keeping its gaze inside a ±2.5‐degree fixation window). After a 500 ms fixation period, an audible go‐signal was given for initiating the grasping movement, which consisted of reaching, grasping, and pulling the object on the plate (holding time: 500–900 ms)[24].

**Saccade test**. In the visually guided saccade task, monkeys had to maintain fixation within a window of 2 × 2 visual degrees around a small green spot in the center of the display for a fixed duration of 450 ms, after which a single green saccade target appeared at one of ten possible positions on the screen, spaced 15 (horizontal) or 11 (vertical) degrees apart. After a variable time, the green fixation spot dimmed, indicating to the animal to saccade towards the target location. The presence of spatially selective saccadic SUA or MUA responses was confirmed using a one-way ANOVA with factor target position (*p* < 0.001 for all target-selective cells).

### Scanning

Functional images were acquired with a 3.0 T full-body scanner (TIM Trio; Siemens), using a gradient-echo T2*-weighted echo-planar imaging (EPI) sequence (40 horizontal slices; TR: 2s; TE: 16 ms; 1.25 mm^3^ isotropic voxels) with a custom-built eight-channel phased-array receive coil, and a saddle-shaped, radial transmit-only surface coil [79]. Before each scanning session, a contrast agent, monocrystalline iron oxide nanoparticle (MION) (Feraheme: AMAG pharmaceuticals; Rienso: Takeda) was injected into the femoral/saphenous vein (7–11 mg/kg) [78].

To verify the stimulation positions, structural MR images (0.6 mm resolution) were acquired in every sedated scan session (prior to the start of the fMRI experiment) while the electrode was located at the exact stimulation site inside a standard recording grid (Crist Instruments, Hagerstown, MD, US). In the few sessions in which the latter could not be achieved, we inserted glass capillaries filled with a 2% copper sulphate solution into the grid at several positions, acquired structural MR images (0.6 mm resolution) and reconstructed the electrode penetrations using SPM 5 (Statistical Parametric Mapping).

In every scanning session, a Platinum/Iridium electrode (impedance 50–200 kΩ in situ, FHC, Bowdoinham, ME) was inserted in the grid through glass capillaries serving as guide tubes (Plastics One Inc, Kent, United Kingdom; FHC, Bowdoinham, ME, US). A platinum wire served as ground. The electrical microstimulation (EM) signal was produced using an eight-channel digital stimulator (DS8000, World Precision Instruments) in combination with a current isolator (DLS100, World Precision Instruments). During stimulation blocks, a single EM train was applied in every trial.

In awake scanning sessions, the animals were either fixating a spot on a screen (Fix) or performing memory-guided saccades (Sacc) towards ten different positions contralateral to the stimulated hemisphere. Briefly, during the memory-guided saccade task a saccade target was flashed for 200 ms on the screen, and the animals had to maintain fixation (300–1,500 ms) until the dimming of the fixation point instructed an eye movement to the remembered target location. During the baseline fixation task (Fix0), only a central fixation point was displayed on the screen, while during the control fixation task (Fix1), one distractor (identical to the saccade target in the Sacc task) was shown on the screen with the same position and timing parameters as the saccade target in the memory saccade task. The color of the fixation point indicated to the animals to either maintain fixation or to make saccades. In this study, the data collected during all three tasks were combined. The three tasks were presented to the animals in blocks, and EM was administered during all three tasks, thus creating six types of blocks which were alternated in one run in pseudo-random order. We alternated between stimulation and no-stimulation blocks (each lasting 40 s), with each run lasting 245 pulses (490 s).

Stimulation trains in awake scan sessions lasted 500 ms and were composed of biphasic square-wave pulses (repetition rate 200 Hz; amplitude 200 μA). Note that pilot experiments showed that a current amplitude of less than 200 μA did not evoke increased fMRI-activations. Each pulse consisted of 190 μs of positive and 190 μs of negative voltage, with 0.1 ms between the two pulses (total pulse duration: 0.48 ms). During sedated scanning sessions, a trial-by-trial stimulation protocol was used similar to the awake sessions (one EM train every 3 s, approximately). EM trains in sedated sessions lasted 250 ms with an amplitude of 1 mA, while other EM-parameters remained similar (200 Hz, 0.48 ms pulse duration). The timing of the EM pulses during the fMRI experiment was computer controlled. Note that pilot experiments showed that a current amplitude of 200 μA (= current strength during awake sessions) during sedated sessions only caused increased fMRI-activations around the tip of the electrode.

### Sedation

During sedated scan sessions, a 0.5/0.5 cc mixture of ketamine (Ketalar; Pfizer) and medetomidine (Domitor; Orion) was administered every 45 min. The animals were video-controlled during sedation, and body temperature was maintained using a heating pad.

### Data Analysis

Off-line image reconstruction was conducted to overcome problems inherent to monkey body motion at 3T. Details about the image reconstruction protocol have been given elsewhere [79]. Briefly, the raw EPI images were corrected for lowest-order off-resonance effects and aligned with respect to the gradient-recalled-echo reference images before performing a SENSE (sensitivity encoding) image reconstruction [80]. Corrections for higher-order distortions were performed using a non-rigid slice-by-slice distortion correction.

Data were analyzed using statistical parametric mapping (SPM5) and BrainMatch software, using a fixed-effect GLM. Realignment parameters were included as covariates of no interest to remove brain motion artifacts. Spatial preprocessing consisted of realignment and rigid coregistration with a template anatomy (M12) [11]. To compensate for echo-planar distortions in the images as well as inter-individual anatomical differences, the functional images were warped to the template anatomy using non-rigid matching BrainMatch software [81]. The algorithm computes a dense deformation field by the composition of small displacements minimizing a local correlation criterion. Regularization of the deformation field is obtained by low-pass filtering. The functional volumes were then resliced to 1 mm^3^ isotropic and smoothed with an isotropic Gaussian kernel (full width at half maximum: 1.5 mm). Single subject and group analyses were performed, and the level of significance was set at *p* < 0.001, uncorrected for multiple comparisons. For display purposes, SPM T-maps were presented on coronal or flattened representations of the M12 anatomical template, using xjView toolbox (http://www.alivelearn.net/xjview) and Caret software (version 5.64; http://brainvis.wustl.edu/wiki/index.php/Caret:About), respectively.

The exact locations and extents of the fMRI-activations were verified on the animal’s own EPI-images. Percent signal change was calculated in regions of interest (ROIs), and statistical significance was tested using MarsBaR (version 0.41.1). We considered a set of 32 ROIs for early visual areas and the ROIs of all brain areas connected to AIP [27], which included premotor, prefrontal, parietal, temporal, and visual ROIs (F5a, F5p, F5c, 45A, 45B, 46v, FEF, AIP, LIP, MIP, CIP, PIP, PFG, STP, OT, PITv, PITd, TE, TEr, FST, MSTv, MT, S2, V1, V2, V3A, V3, V4, V4A, V4T, V6A, V6). Moreover, we also included an additional set of ROIs of frontal areas that are not connected with AIP: F1, F2, F3, F4, F6, and F7. Note that the no-stimulation condition served as the baseline. The significance threshold for one-tailed *t*-tests was set at *p* = 0.05, corrected for multiple comparisons (32 *t*-tests calculated; *p* = 0.05/32 = 0.0016). Standard fMRI analysis methods were used, as described in previous studies [30,52]. All regions of interest were described previously [11,30,62].

To quantify the similarity between the awake and sedated states and between animals, a Pearson correlation was calculated between the percentage of significant voxels (*t*-value > 3.1: *p* < 0.001 uncorrected) per ROI in each state (*awake*-*sedated*) or in each animal, across the set of 32 ROIs of all early visual areas and all areas connected to AIP. The significance of the correlations between animals was calculated using a permutation test, in which the 32 calculated percentages of significantly (*p* < 0.001 uncorrected) activated voxels were randomly assigned (5,000 times) to the 32 ROIs, after which the correlations between corresponding ROIs were calculated. *P*-values were calculated as the proportion of correlations exceeding the actual correlation between corresponding ROIs. Moreover, to confirm the consistency of the activations across animals and states, a conjunction analysis was performed on the data of all animals (at *p* < 0.05 uncorrected for each animal).

## Supporting Information

S1 Fig Electrode locations (blue arrows). Red arrow in the top left panel indicates an electrode track where stereo-selective single unit responses were found for monkey M. For animals T and K, reconstructed electrode positions are shown.
(TIF)

S2 Fig T-score maps for the contrast *EM*-*NoEM*, averaged over all animals and states, represented on coronal sections (template anatomy). A. aAIP-EM. B. pAIP-EM. C: conjunction analysis between *aAIP-EM* and *pAIP-EM* (*p* < 0.01, uncorrected). d. LIP-EM.
(TIF)

S3 Fig T-score maps for the contrast *EM*-*NoEM*, averaged over a (randomly selected) equal number of runs per animal, represented on coronal sections (template anatomy). A. aAIP-EM (14 runs per animal). B. pAIP-EM (eight runs per animal). C. LIP-EM (eight runs per animal).
(TIF)

S4 Fig Contralateral ROIs. Percent signal change calculated in the ROIs contralateral to the stimulated hemisphere; * *p* < 0.05, corrected for multiple comparisons. Vertical black lines indicate standard error of the mean. A. aAIP. B. pAIP. C. LIP.
(TIF)

S5 Fig Summary of EM results for aAIP and pAIP. A. Percent signal change elicited during aAIP-EM (blue) and pAIP (red) for all areas that are anatomically connected to AIP and a set of cortical areas that are not connected to AIP (dashed box). * *p* < 0.05, Bonferroni corrected for multiple comparisons. Upper row of * indicates significance for pAIP-EM; lower row for aAIP-EM. B. Percent activated voxels in the same cortical areas for aAIP-EM (blue) and pAIP-EM (red).
(TIF)

S6 Fig T-score maps for the contrast *EM*-*NoEM*, conjunction analysis over animals and states, (*p* < 0.05 uncorrected), represented on coronal sections (template anatomy). A. aAIP. B. pAIP. C. LIP.
(TIF)

S7 Fig Control sessions. A. Microstimulation of area PFG. T-score maps for the contrast EM-NoEM, represented on coronal sections (template anatomy). B. Microstimulation of area MIP.
(TIF)

S1 Table Overview of stimulation sites. For every animal and stimulated area, the position relative to the IPS-tip is given, together with the neuronal responses in the area. For every animal, state, and stimulated area, the number of runs is given.
(DOCX)

S2 Table Results of two-way ANOVA on predefined ROIs, with factors *stimulation* [*EM*-*NoEM*] and *area* [*aAIP*-*pAIP*] (leftmost columns), [*pAIP*-*LIP*] (rightmost columns). F- and *p*-values are given for the interaction term. df = 1.
(DOCX)

## Abbreviations

AIP: anterior intraparietal area
aAIP: anterior AIP
CIP: caudal intraparietal area
EM: Electrical Microstimulation
FEF: Frontal Eye Fields
fMRI: functional magnetic resonance imaging
FST: fundus of the superior temporal sulcus
IPS: intraparietal sulcus
LGN: lateral geniculate nucleus
LIP: lateral intraparietal area
LOC: Lateral Occipital Complex
MIP: medial bank of the intraparietal sulcus
MT: middle temporal area
MST: medial superior temporal area
MSTv: ventral part of MST
MUA: multi-unit activity
OT: occipitotemporal area
pAIP: posterior AIP
PFG: area in the rostral portion of the inferior parietal lobule
PIP: posterior intraparietal area
PITd: dorsal part of the posterior inferotemporal cortex
PITv: ventral part of the posterior inferotemporal cortex
PMv: ventral premotor cortex
PSC: percent signal change
ROI: region of interest
SFM: structure-from-motion
STS: superior temporal sulcus
SUA: single unit activity
TE: anterior area in inferotemporal cortex
TEO: posterior area in inferotemporal cortex
