# Supplementary figures and images for: Effective Connectivity of Depth-Structure–Selective Patches in the Lateral Bank of the Macaque Intraparietal Sulcus
Source: PLoS Biol. 2015 Feb 17;13(2):e1002072. doi: 10.1371/journal.pbio.1002072 (PMC4331519; doi:10.1371/journal.pbio.1002072)

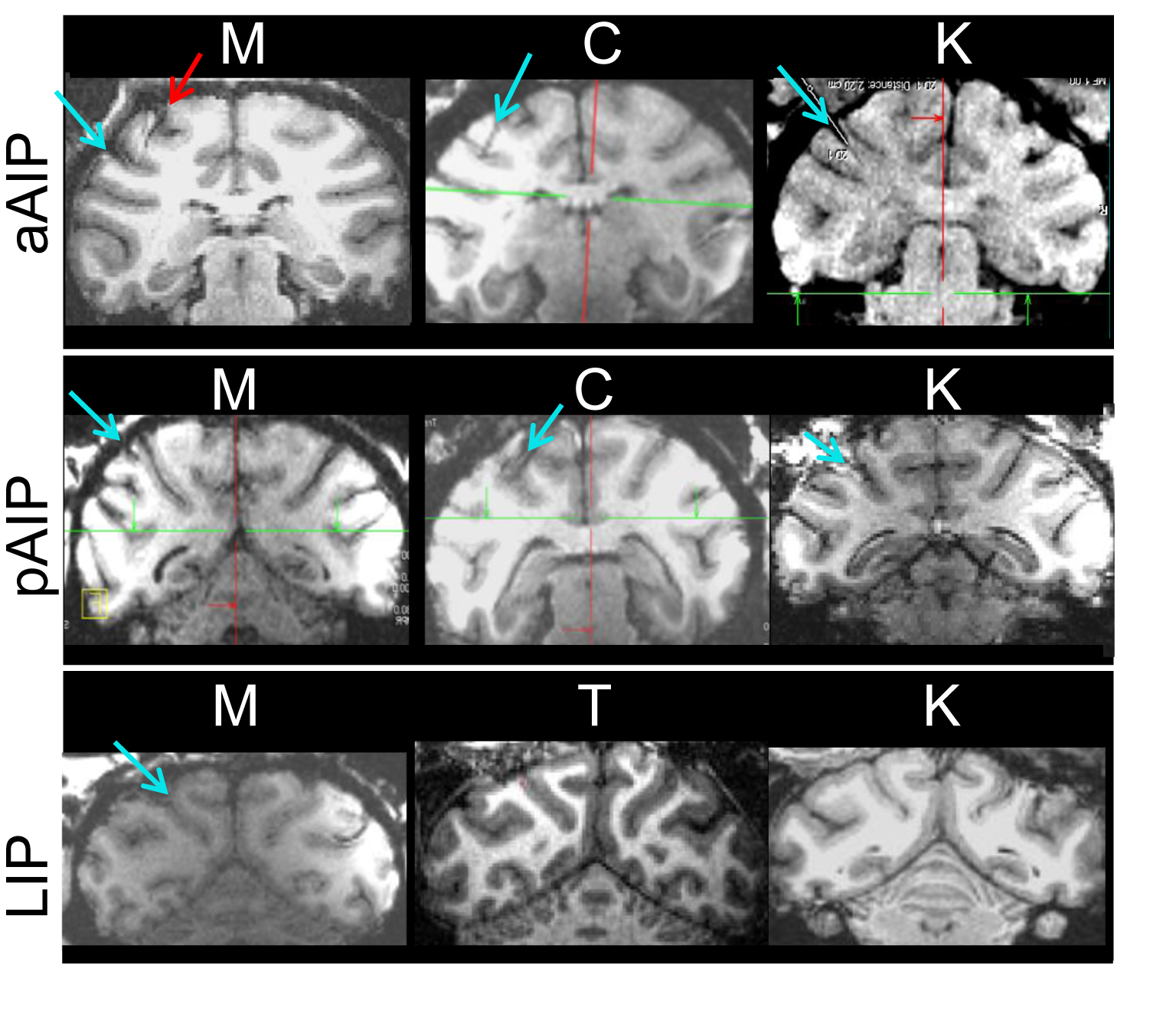

Supplement: S1 Fig — Red arrow in the top left panel indicates an electrode track where stereo-selective single unit responses were found for monkey M. For animals T and K, reconstructed electrode positions are shown. (TIF) [file pbio.1002072.s001.tif]

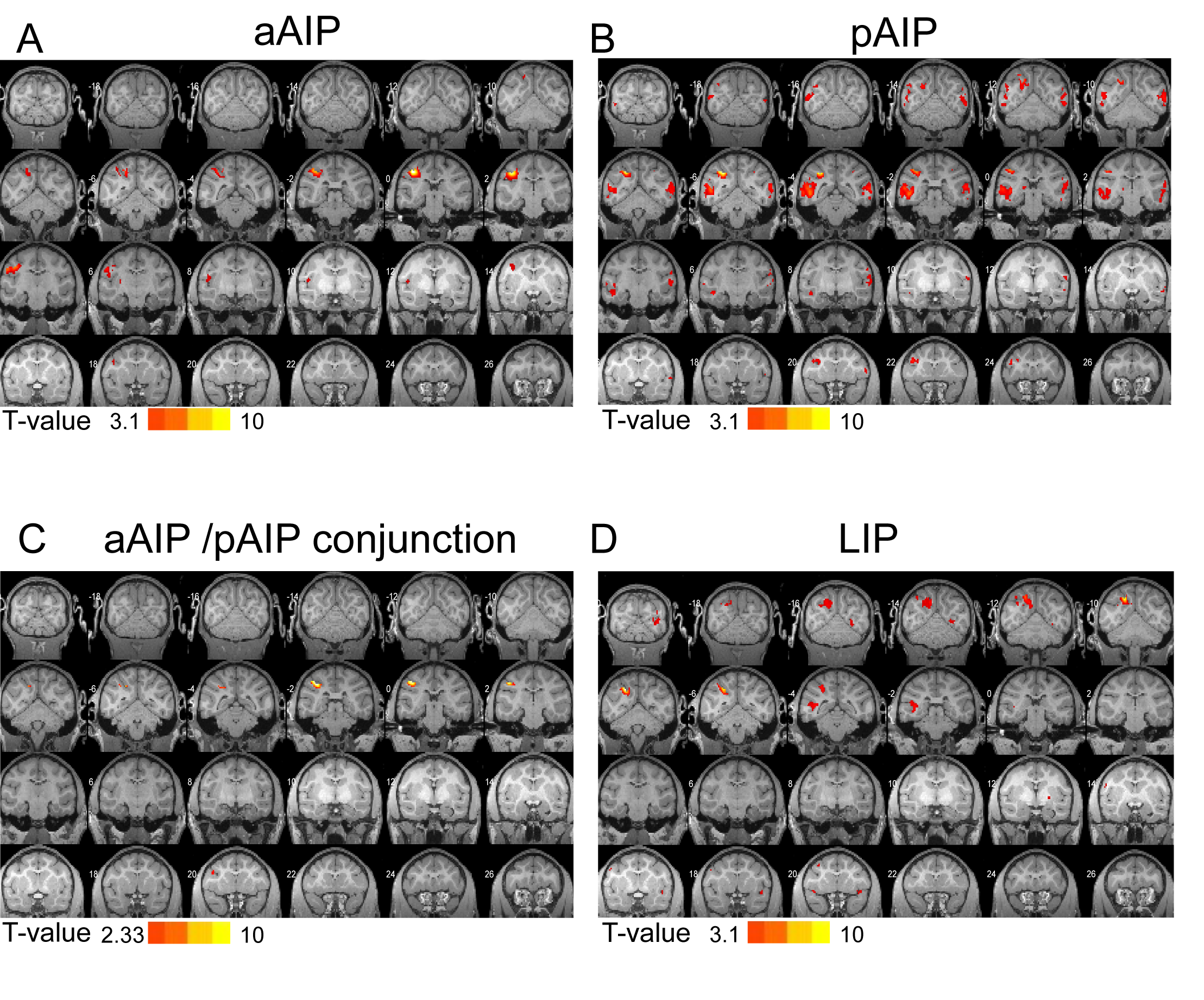

Supplement: S2 Fig — A. aAIP-EM. B. pAIP-EM. C: conjunction analysis between aAIP-EM and pAIP-EM (p < 0.01, uncorrected). d. LIP-EM. (TIF) [file pbio.1002072.s002.tif]

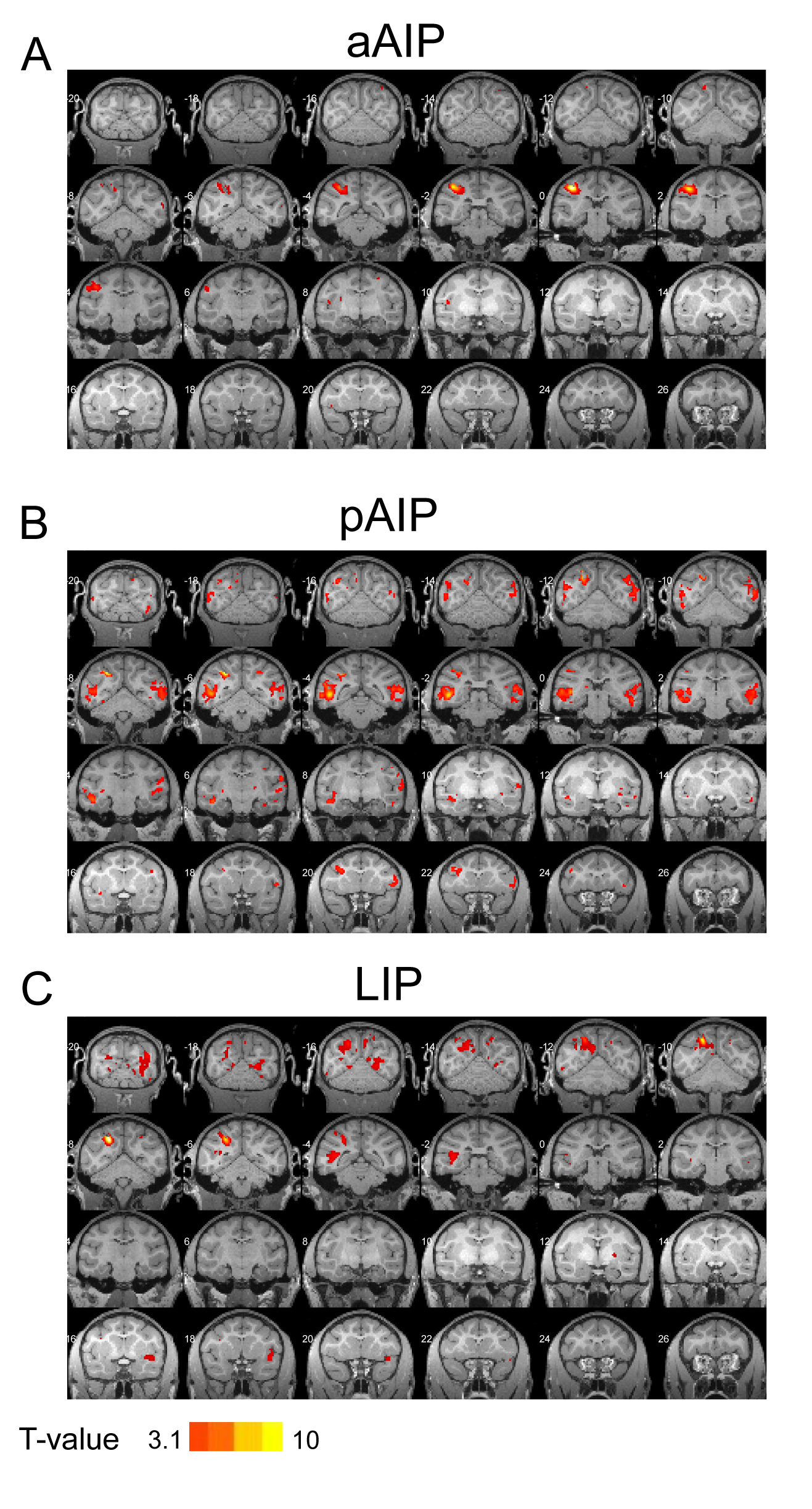

Supplement: S3 Fig — A. aAIP-EM (14 runs per animal). B. pAIP-EM (eight runs per animal). C. LIP-EM (eight runs per animal). (TIF) [file pbio.1002072.s003.tif]

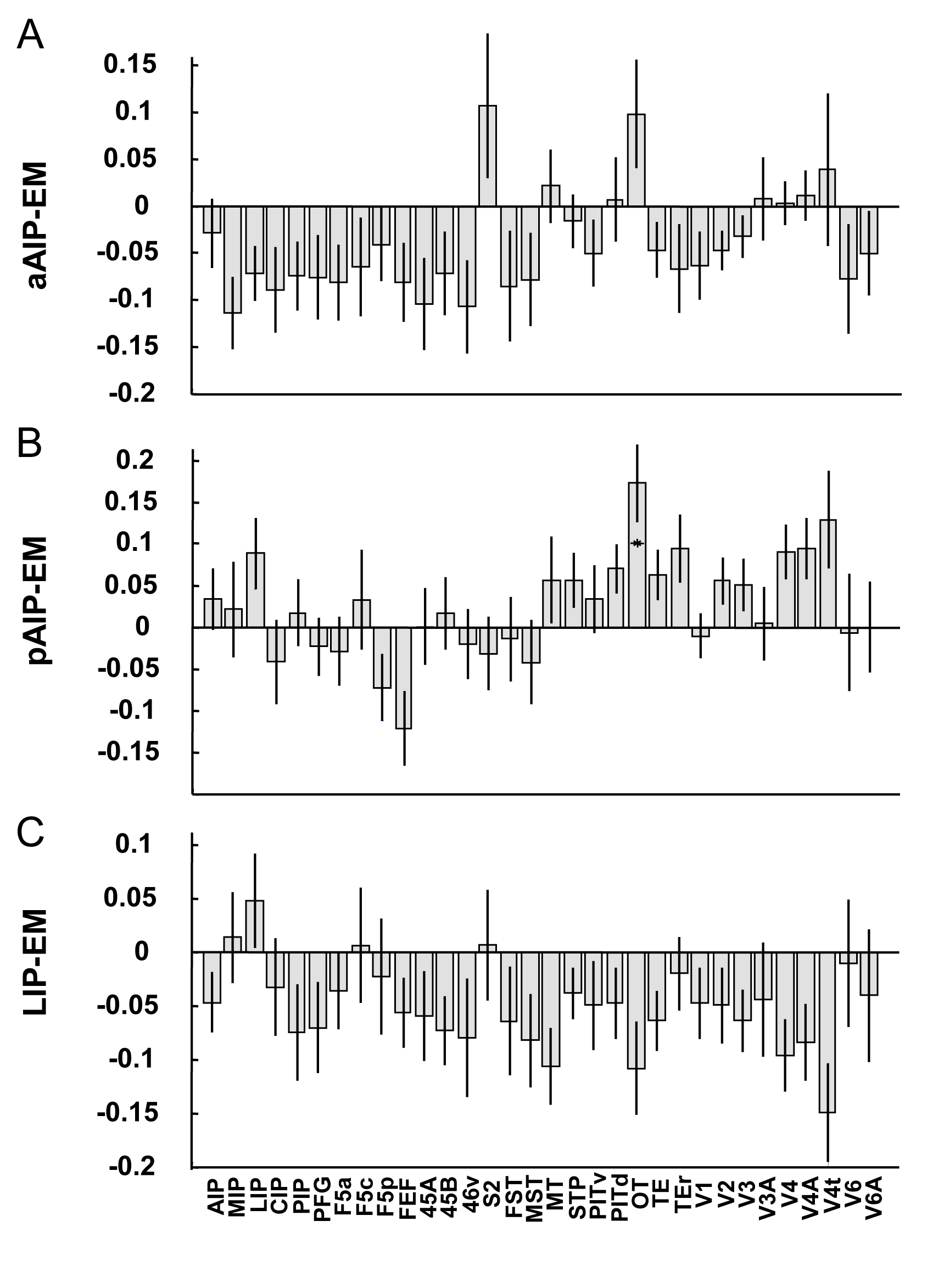

Supplement: S4 Fig — Vertical black lines indicate standard error of the mean. A. aAIP. B. pAIP. C. LIP. (TIF) [file pbio.1002072.s004.tif]

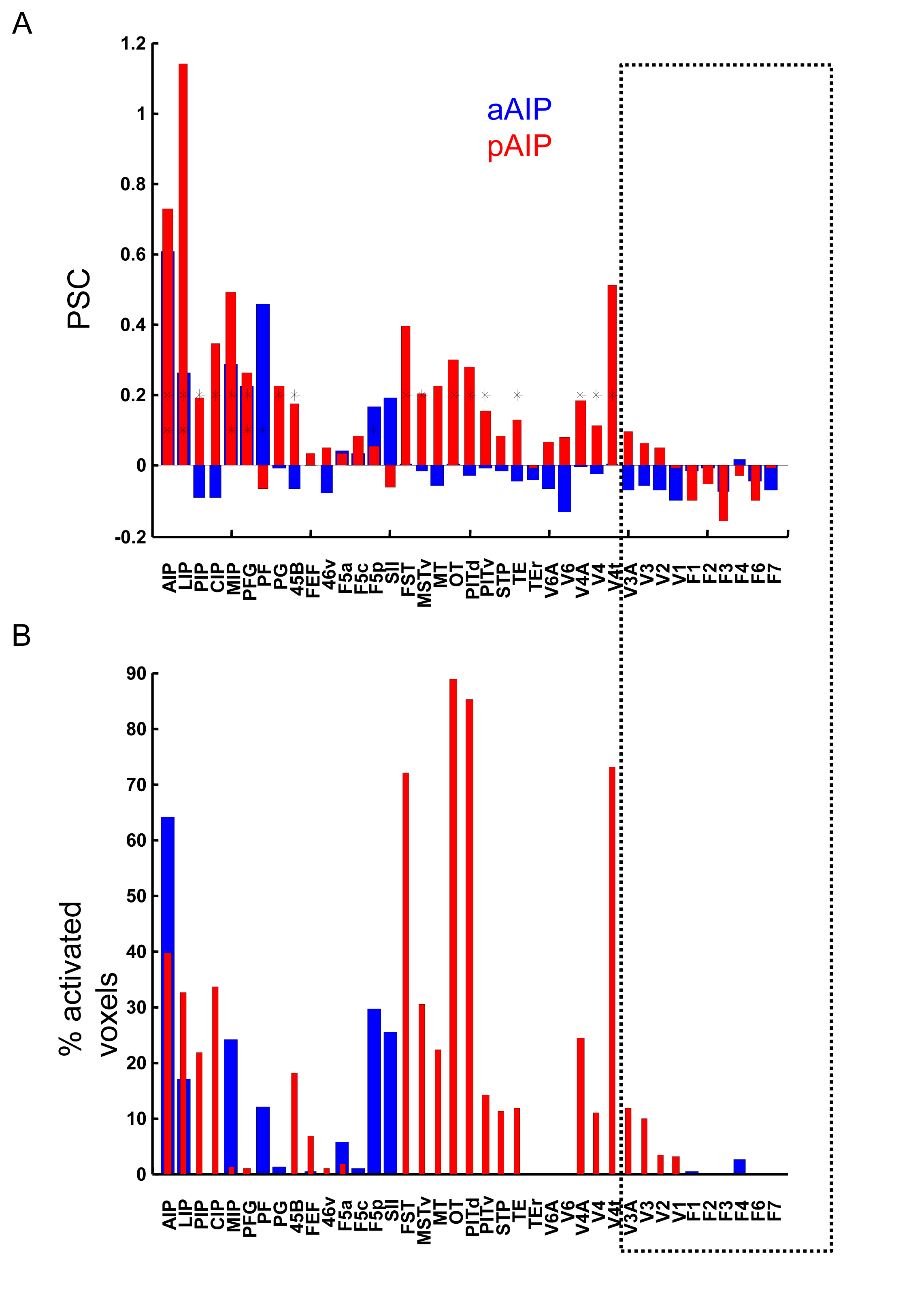

Supplement: S5 Fig — A. Percent signal change elicited during aAIP-EM (blue) and pAIP (red) for all areas that are anatomically connected to AIP and a set of cortical areas that are not connected to AIP (dashed box). * p < 0.05, Bonferroni corrected for multiple comparisons. Upper row of * indicates significance for pAIP-EM; lower row for aAIP-EM. B. Percent activated voxels in the same cortical areas for aAIP-EM (blue) and pAIP-EM (red). (TIF) [file pbio.1002072.s005.tif]

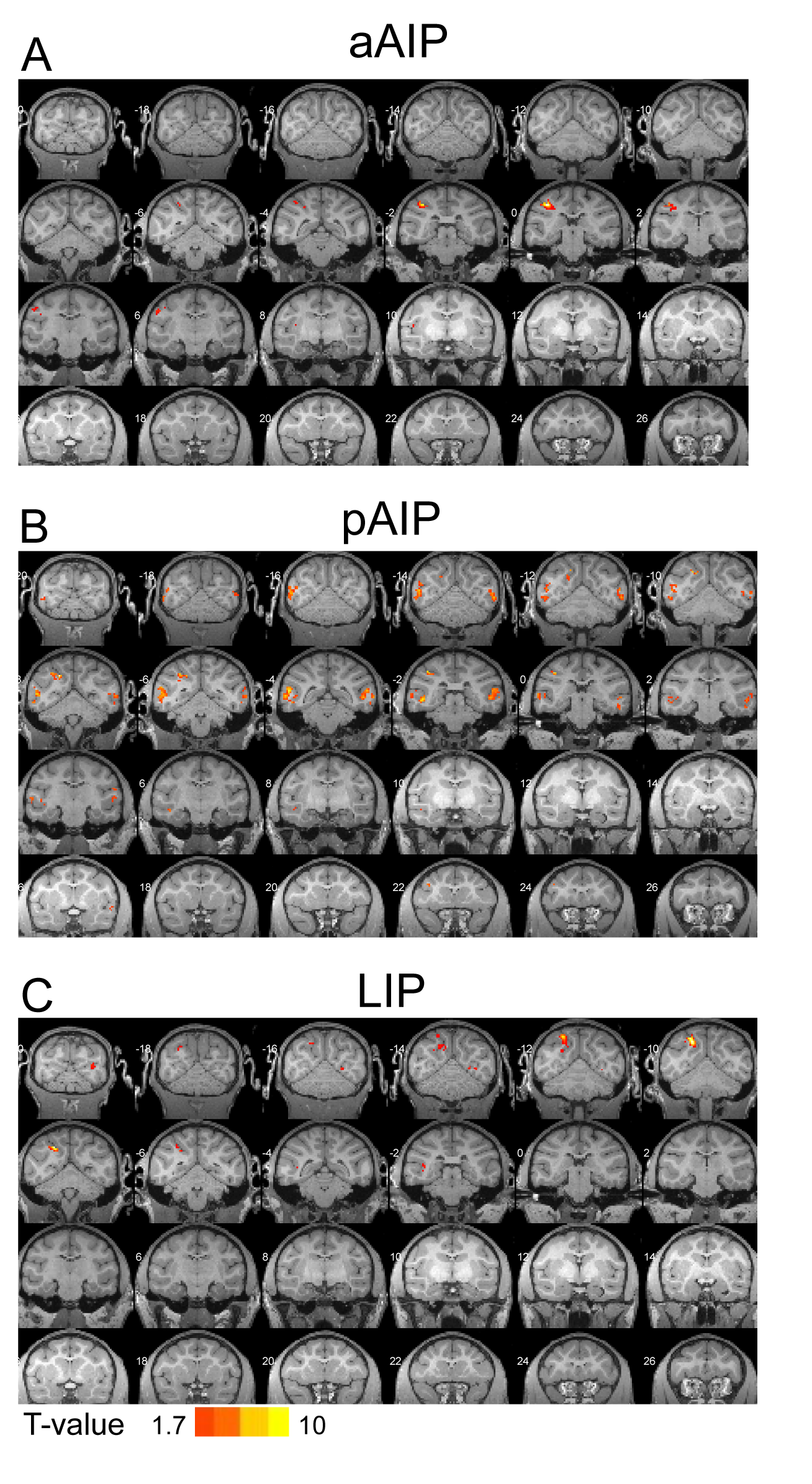

Supplement: S6 Fig — A. aAIP. B. pAIP. C. LIP. (TIF) [file pbio.1002072.s006.tif]

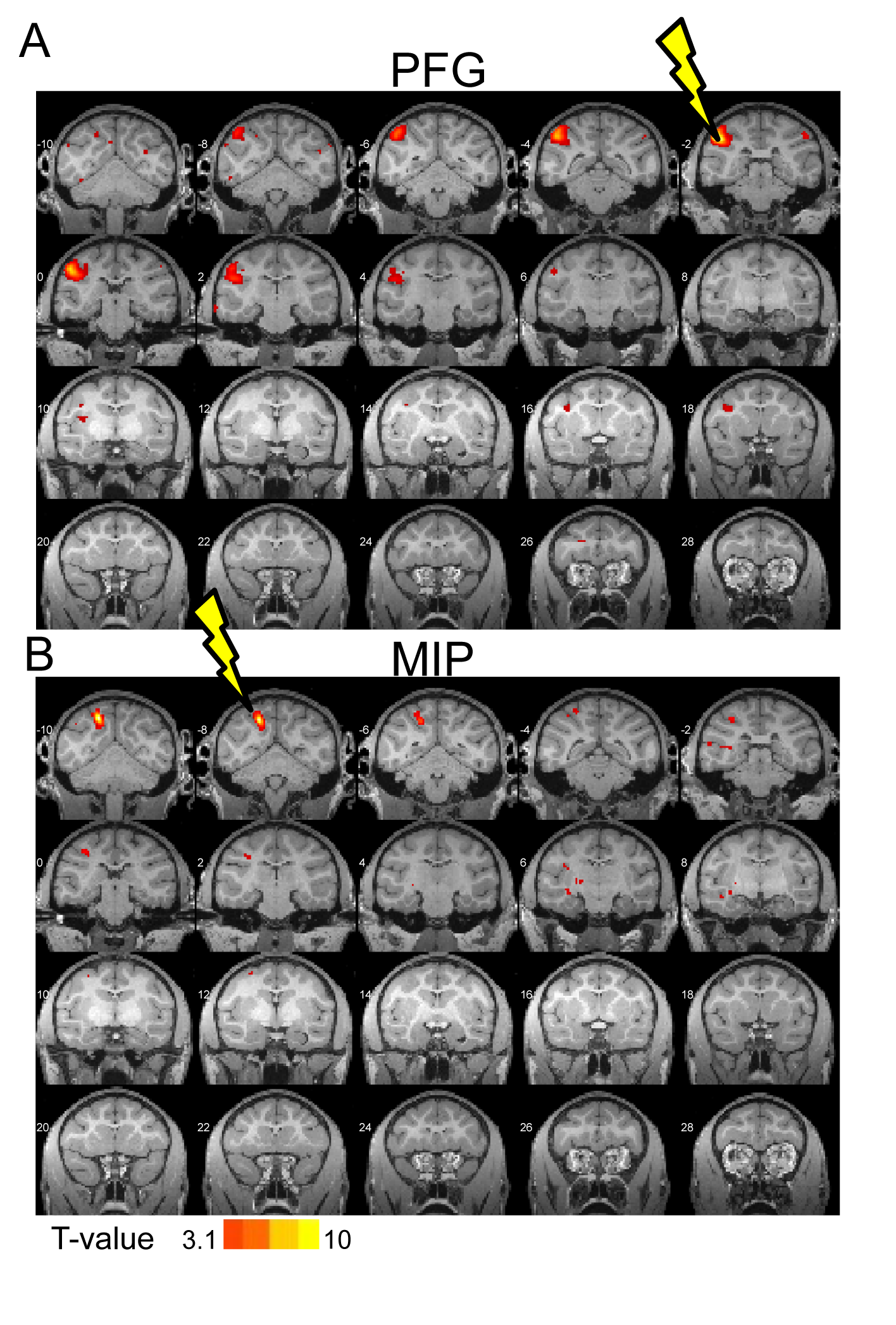

Supplement: S7 Fig — A. Microstimulation of area PFG. T-score maps for the contrast EM-NoEM, represented on coronal sections (template anatomy). B. Microstimulation of area MIP. (TIF) [file pbio.1002072.s007.tif]
