# Supplementary material for: Effective Connectivity of Depth-Structure–Selective Patches in the Lateral Bank of the Macaque Intraparietal Sulcus
Source: PLoS Biol. 2015 Feb 17;13(2):e1002072. doi: 10.1371/journal.pbio.1002072 (PMC4331519; doi:10.1371/journal.pbio.1002072)
Supplement: S1 Table — For every animal and stimulated area, the position relative to the IPS-tip is given, together with the neuronal responses in the area. For every animal, state, and stimulated area, the number of runs is given. (DOCX) [file pbio.1002072.s008.docx]

|  | M | K | C | T |
| --- | --- | --- | --- | --- |
| aAIP | IPS-tip – 7 mm | IPS-tip – 6 mm | IPS-tip – 6 mm |  |
|  | Stereo  17 awake  41 sedated | Stereo  14 awake | 15 sedated |  |
| pAIP | IPS-tip – 14 mm | IPS-tip – 9 mm | IPS-tip – 9 mm |  |
|  | -Objects  -Stereo  23 awake  7 sedated | -Objects  -Stereo  8 sedated | -Objects  -Grasping  23 sedated |  |
| LIP | IPS-tip – 17 mm | IPS-tip – 14 mm |  | IPS-tip – 15 mm |
|  | Saccades  25 awake | Saccades  8 awake |  | Saccades  27 awake |

Table S1. Overview of stimulation sites. For every animal and stimulated area, the position relative to the IPS- tip is given, together with the neuronal responses in the area. For every animal, state and stimulated area, the number of runs is given.
