# Supplementary material for: Effective Connectivity of Depth-Structure–Selective Patches in the Lateral Bank of the Macaque Intraparietal Sulcus
Source: PLoS Biol. 2015 Feb 17;13(2):e1002072. doi: 10.1371/journal.pbio.1002072 (PMC4331519; doi:10.1371/journal.pbio.1002072)
Supplement: S2 Table — F- and p-values are given for the interaction term. df = 1. (DOCX) [file pbio.1002072.s009.docx]

|  | **aAIP*pAIP** |  | **pAIP * LIP** |  |
| --- | --- | --- | --- | --- |
|  | F | p | F | p |
| v1 | **5.72** | **0.02** | 1.8 | 0.18 |
| V2 | **9.79** | **0.002** | **4.08** | **0.04** |
| V3 | **8.55** | **0.004** | **5.73** | **0.02** |
| V3A | **6.22** | **0.01** | 0.55 | 0.46 |
| V4t | **49.35** | **1.5e-11** | **22.58** | **3.3e-6** |
| v4 | **11.54** | **7.7e-4** | **6.18** | **0.01** |
| v4a | **13.97** | **2.2e-4** | **13.95** | **2.3e-4** |
| V6 | **6.35** | **0.01** | 0.31 | 0.58 |
| V6A | 2.42 | 0.12 | 0.1 | 0.33 |
| OT | **33.81** | **1.6e-8** | 25.06 | 1.01 |
| PITd | **52.05** | **4.68e-12** | 14.25 | 1.98 |
| PITv | **11.13** | **9.6e-4** | **17.09** | **4.8e-5** |
| TE | **16.38** | **6.7e-5** | **14.02** | **2.2e-4** |
| TEr | 0.28 | 0.60 | 0.19 | 0.66 |
| FST | **28.36** | **2e-7** | **6.35** | **0.01** |
| MT | **15.90** | **8e-5** | **9.2** | **0.003** |
| MST | **8.54** | **0.004** | 1.729 | 0.19 |
| STP | **5.88** | **0.02** | **4.78** | **0.03** |
| S2 | **7.76** | **0.006** | 0.00018 | 1 |
| MIP | **5.76** | **0.02** | 0.96 | 0.33 |
| LIP | **74.58** | **3.8e-16** | 0.76 | 0.38 |
| CIP | **37.87** | **2.5e-9** | 0.46 | 0.5 |
| PIP | **19.68** | **1.3e-5** | 2.50e-4 | 0.987 |
| PFG | 0.23 | 0.63 | 3.04 | 0.08 |
| AIP | 1.47 | 0.23 | **54.59** | **1.9e-12** |
| 45B | **14.04** | **2.2e-4** | **12.52** | **4.7e-4** |
| 45A | **4.45** | **0.04** | 0.68 | 0.41 |
| 46v | 3.32 | 0.07 | 0.40 | 0.53 |
| FEF | 0.23 | 0.63 | 0.59 | 0.44 |
| F5p | 1.95 | 0.16 | 1.82 | 0.18 |
| F5a | 0.025 | 0.87 | 0.32 | 0.57 |
| F5c | 0.387 | 0.534 | 0.30 | 0.58 |

Table S2. Results of 2-way ANOVA on predefined ROIs, with factors *stimulation* [*EM*-*NoEM*] and *area* [*aAIP*-*pAIP*] (leftmost columns), [*pAIP*-*LIP*] (rightmost columns) . F- and p-values are given for the interaction term. df = 1.
